# The Role of AChE in Swimming Behavior of* Daphnia magna*: Correlation Analysis of Both Parameters Affected by Deltamethrin and Methomyl Exposure

**DOI:** 10.1155/2017/3265727

**Published:** 2017-10-19

**Authors:** Qing Ren, Ruibin Zhao, Cheng Wang, Shangge Li, Tingting Zhang, Zongming Ren, Meiyi Yang, Hongwei Pan, Shiguo Xu, Jianping Zhu, Xun Wang

**Affiliations:** ^1^Institute of Environment and Ecology, Shandong Normal University, Jinan 250014, China; ^2^Management College, Ocean University of China, Qingdao 266100, China; ^3^Department of Electrical and Computer Engineering, Tandon School of Engineering, New York University, Brooklyn, NY 11201, USA

## Abstract

The unpredictable toxicity of insecticides may cause behavior disorder of biological organisms. In order to assess the role of acetylcholinesterase (AChE) in swimming behavior of* Daphnia magna*, a correlation analysis of both parameters in 24 h exposure of deltamethrin (DM) and methomyl (MT) was investigated. The behavior responses of* D. magna* in DM (13.36 *μ*g/L and 33.40 *μ*g/L) and MT (19.66 *μ*g/L and 49.15 *μ*g/L) suggested that recovery behavior in the adjustment phase was crucial, and behavior homeostasis provided them with an optimal way to achieve a wider tolerance against environmental stress. During the experiment, positive effects on AChE activity occurred in the beginning of the exposure. Even though the de novo synthesis of AChE in* D. magna* might help it recover, the AChE inhibition in different treatments could be observed. Some induction effects on AChE activity at the beginning of exposure occurred, and a 50% decrease may cause toxic effects on behavior. In most treatments, the results showed that both behavior strength and AChE activity stayed in the same field within a correlation circle. These results illustrated that the environmental stress caused by both DM and MT could inhibit AChE activity and subsequently induce a stepwise behavior response, though both pesticides affect it as direct and indirect inhibitors, respectively.

## 1. Introduction

Numerous pesticides pose incidental threats to nontarget organisms by creating environmental pollution and damaging people's health. Pyrethroids and carbamate pesticides have been used throughout the world to control pests in agricultural crops, forests, and wetlands [1–3]. However, the extensive utilization of these insecticides may impair biological communities, inducing an unbalance in aquatic ecosystems, and cause unpredictable toxicity to humans and numerous other biological organisms. According to the law of tolerance [4–6], once the quantity or quality of one factor exceeds the toxicity threshold, the growth and reproduction of organisms will be limited. Therefore, these insecticides can exert their toxicity to organisms as limiting factors. If even one environmental factor changes and limits organisms, this can drive others to compensate and strengthen the accommodation in the fluctuating habitat [7]. For instance, recognition of burrow's olfactory signature was an efficient discrimination mechanism when light becomes a limiting factor in the dark [8].

The adaptation of individuals in a polluted aquatic environment is imperative to their own survival. Homeostasis is a set of processes to achieve and maintain a state of dynamic equilibrium. It is a way to maintain internal stability and appropriate responses to both internal and external stimuli [9]. Behavior homeostasis is proposed as a neutral term that can be applied across phylogeny from aneural single-celled protozoa to complex mammals when discussing issues related to stimulus detection and assessment. Behavior is being used in its broadest sense to include any measurable and observable response to an iterative stimulus across phylogeny [10]. A behavior homeostasis mechanism provides a perfect way for aquatic individuals to develop an ability to tolerate a wider variety of environmental limiting factors [11], and this behavior response to environment pollution is an adaptive process: aquatic organism can swim from polluted environment to clean one based on their instinct: Avoidance Behavior [12]. Previous research has suggested that aquatic organisms have the ability to adapt to the aquatic environmental stress by adjusting the tolerance of their limiting factors, which might induce a stepwise behavior response including acclimation, adjustment, and so on [13]. Behavior response of different aquatic organisms under environmental stress has been reported to be sensitive to sublethal chemical concentrations, such as crustaceans [14, 15], snails [16], insects [17], and fish [18]. Meanwhile, movement changes are suitable indicators in the ecological risk assessment [19] and behavior monitoring is reported as a useful means for toxicity checking [20]. Furthermore, there have been many investigations on the intrinsic mechanisms of the behavior response under environmental stress, for example, the hormonal levels [21], the acetylcholinesterase activity in brain [22], dysregulation of the right brain [23], and the modulating covariation in physiological traits [24].

Previous research suggested that deltamethrin (DM) and methomyl (MT) are two kinds of pesticides with different toxic mechanisms. DM is a synthetic type II pyrethroid [25]. It could inhibit ATPase in synaptosomal membrane, which will cause the accumulation of neurotransmitter AChE, and then induce the toxic effect. It is known to be toxic to diverse aquatic organisms because of the effects it has on the nervous system, which is involved in signal transduction and the proteome regulation as registered [26, 27]. MT is a commonly used monomethyl carbamate insecticide to control a wide range of insects and spider mites through direct contact and ingestion [28]. MT may also exert its toxic effect on nontarget organisms, by inducing an oxidative stress that alters in enzymatic and nonenzymatic antioxidant systems [29, 30].

Acetylcholinesterase (AChE) is a key enzyme that hydrolyzes the neurotransmitter acetylcholine in cholinergic synapses of both vertebrates and invertebrates; this may affect a nerve's ability to conduct due to the accumulation of acetylcholine in the body once AChE is inhibited [31–33]. Behavior movement of organisms is related directly to nerve conduction [34]. Swimming behavior of aquatic organisms would benefit predation, antipredation, and avoidance abilities, which could increase the survival chance of these organisms in their aquatic environment. Therefore, the inhibition of AChE would decrease the ability of behavior homeostasis [35], potentially inducing a higher survival risk.

*Daphnia magna*, a small planktonic invertebrate crustacean (0.5–5.0 mm) with a short life cycle, is very sensitive to environmental changes [36, 37].* D. magna* is a standard organism for toxicity tests, and the species has often been used in bioassays and environmental monitoring of aquatic systems due to the ease and the low economical cost of maintaining cultures [38]. Behavior responses of* D. magna* to environmental stress have been reported [39–41], and there have been a multitude of researches on the toxic endpoint at different levels, for example, individual growth and reproductivity [42], embryonic development and sex differentiation [42], acetylcholinesterase activity [43], and cellular and molecular level [44]. Lovern et al. [45] have investigated the effects of different chemicals on both behavior and physiological changes of* D. magna*, Jensen et al. [46] have established a cause-effect relationship between acetylcholinesterase inhibition and altered locomotor behavior in the carabid beetle, Sismeiro-Vivas et al. [47] have investigated the short-term effects of quirlan on the behavior and acetylcholinesterase activity of* Gambusia holbrooki*, and Tilton et al. [48] have made relationship analysis between AChE inhibition and behavior in zebrafish exposed to copper or chlorpyrifos separately or as mixtures. However, there has been hardly any study on the correlation analysis between the online behavior responses and the continuous AChE inhibition level.

We have focused our study on the behavior response of aquatic organisms after acute exposure, because behavior homeostasis can provide them with a perfect way to have wide tolerance ability against environmental limiting factors. However, there is no direct evidence to clearly show the intrinsic mechanisms of behavior homeostasis yet. We hypothesize that AChE acts as a dominant factor in nerve conduction for swimming behavior within different chemicals that may directly or indirectly inhibit AChE activity continuously; then the aim of this study is to (i) investigate the swimming behavior and AChE inhibition of* D. magna* under the stress of two kinds of pesticides with different toxic mechanisms that act as indirect (DM) and direct (MT) inhibitors of AChE [34], (ii) reveal the relationship between AChE inhibition and behavior responses based on correlation analysis, and (iii) discuss the role that AChE plays in behavior homeostasis.

## 2. Materials and Methods

### 2.1. Materials

The experimental* D. magna* (24 h young) were cultured in our laboratory for more than three generations. The culture was maintained at 20 ± 2°C under a 16 h light : 8 h dark photoperiod (illumination ranged between 3000 and 4500 lx). Culture medium was prepared according to the components of the Standard Reference Water (SRW) [49] and* D. magna* were fed* Scenedesmus obliquus* two times a day. Before feeding* D. magna*, the culture medium of the algae was filtered and then diluted by SRW until the concentration reached 1 × 10^5^ cells/ml. The quantity of the algae was approximately 1% beaker volume. Before the exposure experiments, the gravid female* D. magna* already carrying eggs were removed and cultured individually in 50 ml glass beakers of SRW until they oviposited. The healthy and uninjured neonates were then taken and used for this experiment.

MT and DM were purchased from J&K Chemical Ltd (Beijing). All compounds were of technical grade (95% purity). Stock solutions (stored at 4°C until use), each having a proper concentration, were prepared in dimethyl sulfoxide to make each test solution. The concentration of dimethyl sulfoxide in water was less than 0.5% in all the experiments. Studies showed that dimethyl sulfoxide of such concentration would neither lead to acute toxicity to* D. magna* nor affect the mobility of* D. magna* [50, 51]. Acetylthiocholine iodide (ATCh), 5,5-dithio-2,2-nitrobenzoic acid (DTNB), Sephadex G-25, bovine serum albumin (BSA), and Triton X-100 were purchased from Sigma (Sigma-Aldrich Corporation, St. Louis, MO, USA). All of these chemicals were of analytical grade (95% purity).

### 2.2. Experimental Setup

We set the logarithm spacing concentration of the insecticide in the exposure experiment to measure LC_50_-24 h (50% lethal concentration) of juveniles (48 h young). According to the previous report, LC_50_-24 h of DM on* D. magna* ranged from 0.113 *μ*g/L to 9.4 *μ*g/L [42, 52–54], and MT on* D. magna* was from 7.3 *μ*g/L to 20.0 *μ*g/L [55], so the concentrations of DM (0.113 *μ*g/L, 0.341 *μ*g/L, 1.031 *μ*g/L, 3.113 *μ*g/L, and 9.400 *μ*g/L) and MT (7.30 *μ*g/L, 9.39 *μ*g/L, 12.08 *μ*g/L, 15.55 *μ*g/L, and 20.0 *μ*g/L) were used to conduct the acute toxicity tests. During the experiment,* D. magna* were fed nothing and were placed in three replicates per concentration with 20 young fleas per treatment. This experiment was made in the same condition of culture.

The swimming behavior of* D. magna* was monitored by an online system built in the Research Center for Eco-Environmental Science, Chinese Academy of Sciences [56]. Six test* D. magna* were placed in each flow-through test chamber (3 cm long, 2 cm in diameter), which was closed off on both sides with nylon nets (250 *μ*m), and 3 replicates per concentration were used. A pair of electrodes located on the walls of the test chambers sent a high-frequency signal of alternating current, which was then received by a second pair of noncurrent-carrying electrodes. The behavior strength of all* D. magna* in each test chamber, which was used to show swimming intensity, was transformed by an A/D transformer and the signal changes formed by the A/D transformer were analyzed automatically by software installed on the equipment. The behavior strength was sampled automatically by the system every second, and the average behavior strength data were taken twice an hour in the first two hours and after that once every hour to analyze the trends found in different treatments. Behavior strength that changed from 0 (losing the ability of movement) to 1 (full behavior express) was introduced to illustrate the behavior response differences of* D. magna* [13]. Exposure concentrations were made based on the results of acute toxicity (LC_50_-24 h), and 2 × LC_50_ and 5 × LC_50_ treatments were chosen.

In vivo, AChE inhibition tests were performed by exposing juveniles to several treatments (0,2 × LC_50_ and 5 × LC_50_) in 5000 ml beaker. In the analysis of AChE activity, a 16 h light : 8 h dark photoperiod was applied for 24 h exposure at 21–23°C. We applied three replicates in this experiment with 500 individuals in 2 × LC_50_ and 5 × LC_50_ exposure and 270 individuals in control. No food was added during the experiments. Samples (10 from each treatment) were taken once an hour in the first 4 h and after that once every 2 h in treatments. Once the individual number of* D. magna* that sunk to the bottom of the beaker (not dead) is higher than 10, AChE activity of these individuals was also detected. Once there were less than 10 live test individuals, the AChE experiment stopped. The death of* D. magna *was defined as the inability to swim for more than a few strokes for 15 s after gentle agitation of the test vessel [57].

81 ml 0.1 M disodium hydrogen phosphate and 19 ml 0.1 M sodium dihydrogen phosphate were mixed and then diluted with deionized water to 100 ml to get phosphate buffer (0.1 M, pH 7.4). Homogenates were prepared in an ice-cold phosphate buffer using mechanically driven Teflon fitted Potter-Elvehjem homogenizer for 2 mins at 3000 r.p.m. in ice till total disruption of water fleas. The homogenates were then centrifuged at 12,000 ×g for 20 min at 4°C [58]. The supernatant was used as an enzyme source for measuring the activity of AChE. AChE activity in the homogenates was detected as follows: 50 *μ*L enzyme and 50 *μ*L ATCh (5 mM final concentration) were incubated at 30°C for 15 min in a final volume of 0.1 mL, and then the reaction was stopped by 0.125 mM DTNB-phosphate-ethanol reagent inside 0.9 ml (12.4 mg of DTNB dissolved in 125 mL 95% ethanol, 75 mL distilled water, and 50 mL 0.1 M phosphate buffer, pH 7.5) as the thiol indicator. The color was detected immediately at 412 nm using an ELIASA (Infinite M200) [59]. Based on the Bradford Protein Assay of the protein concentration of enzymatic extracts [60], the AChE activity was detected, which was in unit of nmol/min·mg. The whole-body AChE activity (% of control) was used to analyze the effects of different chemicals on the AChE activity.

### 2.3. Data Analysis

The 50% lethal concentration (LC_50_) values were calculated by probit analysis in the MATLAB 2009 (© 1984–2009 The MathWorks, Inc.). In order to assess the role of AChE in swimming behavior of* Daphnia magna* in different treatments, the average values of three replicates of both the behavior data and whole-body AChE activity (% of control) were calculated and then analyzed based on the linear fitting in MATLAB. The Kalman filtering with linear regression analysis was used in the continuous changes over the 24-hour period of both whole-body AChE activity (% of control) and behavior strength of* D. magna* in different treatments with 95% confidence bounds to eliminate the influence of the environmental noise (Fileds et al., 1991). Linear regression equations of AChE activity (% of control) and behavior strength could be calculated with different constants in different treatments, which might be applied in the toxic effect analysis. After Kalman filtering with linear regression with *p* < 0.05, Principal Component Analysis (PCA) was subsequently used to illustrate the correlation between the inhibition degree of AChE and the behavior responses of* D. magna*. The learning process of PCA was conducted using the Self-Organizing Map toolbox developed by the Laboratory of Information and Computer Science, Helsinki University of Technology in MATLAB environments [61]. One-way analysis of variance (ANOVA) was performed to compare the whole-body AChE activity (% of control) and behavior strength in different behavior stages with different significance (*p* < 0.01 and *p* < 0.05) based on the Stepwise Behavior Response Model [13].

## 3. Results and Discussion

### 3.1. Acute Toxicity

According to the chemical toxicity to the aquatic organism [55], DM and MT had high toxicity to* D. magna*. The acute toxic effects of DM and MT on* D. magna* are shown in Table 1 after probit analysis in MATLAB with 95% confidence interval. The measured LC_50_-24 h values of DM and MT on* D. magna* were 6.68 *μ*g/L and 9.83 *μ*g/L. According to the previous report, 24 h acute ecotoxicity data (as LC 50) ranged from 0.113 *μ*g/L to 9.4 *μ*g/L [42, 52–54], and MT on* D. magna* was from 7.3 *μ*g/L to 20.0 *μ*g/L [55]. These results suggested that the measured LC_50_-24 h values in this study were acceptable.

### 3.2. The Toxic Effects of DM and MT on Both Swimming Behavior and AChE Activity

**Figure 1:**
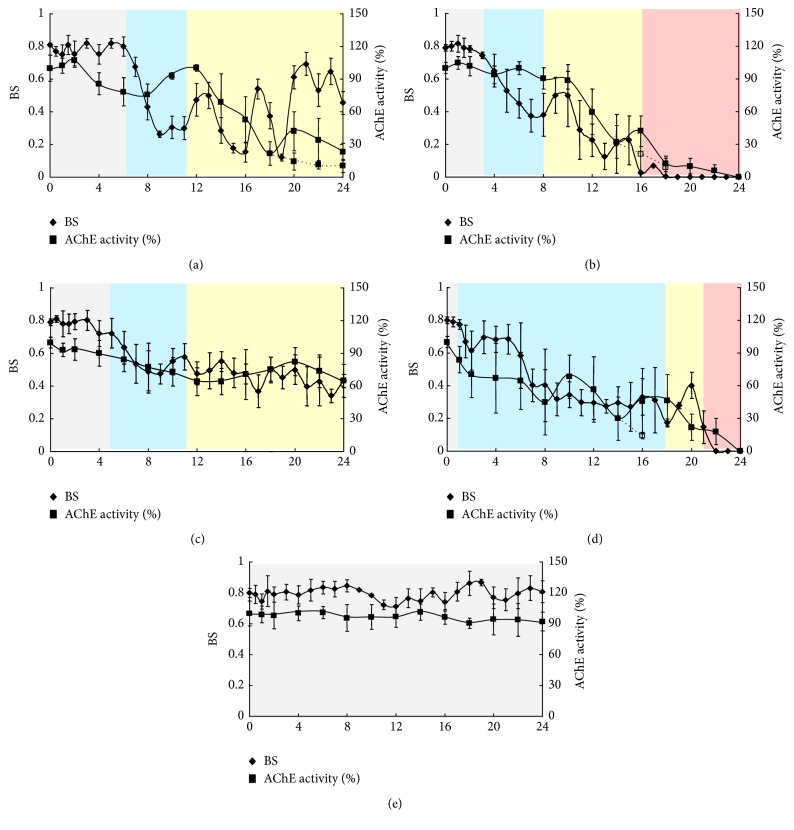
The swimming behavior and the AChE activity (% of control) inhibition of* D. magna* after in vivo exposure in both DM and MT. M ± SD was applied. (a, b, c, d, and e) Showing the behavior responses and AChE activity of* D. magna* in 13.36 *μ*g/L DM, 33.40 *μ*g/L DM, 19.66 *μ*g/L MT, 49.15 *μ*g/L MT, and control, separately. AChE activity of* D. magna* in control at the beginning of the experiments is regarded as 100%. The solid line with round dots represented the behavior responses of* D. magna.* The solid line with triangles represented the AChE activity of live* D. magna* and the dotted line with triangle represented the AChE activity of dead* D. magna* once there were some individuals that died during the exposure periods. Gray shadows mean no effects and stimulation, blue shadows mean the acclimation, yellow shadows mean the (re)adjustment, and red shadows mean the toxic effect.

The swimming behavior and the AChE activity of* D. magna* under the exposure to both DM and MT is shown in Figure 1. Different color shadows mean the different stages based on the Stepwise Behavior Response Model: no effects and stimulation stand for the exposure period before the first significant decrease of behavior strength (SD-BS) occurred (gray shadows) (20%, [13]), acclimation is the period after the first SD-BS until the first adjustment (20% behavior strength increase) (blue shadows), (re)adjustment is from the first adjustment to toxic effects (yellow shadows), and toxic effect starts from the time when behavior strength of* D. magna* is lower than 0.2 and there is no recovery (red shadows).

The average behavior strength in control changed from 0.87 to 0.72 and the average AChE activity was around 100% (from 88% to 105%) (Figure 1(e)). As it is reported by Ren et al. [62], the behavior responses of* D. magna*, which have a fluctuation of 36%, are more intensive than the AChE activity (less than 20% fluctuation) under the DDVP exposure. These suggest that both the swimming behavior and the AChE activity of* D. magna* exposed to DM and MT are relatively stable in control group based on the data from each time point.

However, the swimming behavior in other treatments was inhibited. Under 13.36 *μ*g/L DM exposure (Figure 1(a)), behavior strength showed fluctuating changes. In the first 6 h, it maintained approximately 0.8, and from the 6th to the 9th hour, a drastic decrease occurred from 0.8 to less than 0.3. There were more than 3 behavior adjustments 9 h later, which happened from 11th to 15th, 16th to 19th, 19th to 24th hour, separately according to the adjustment criteria in our previous research (yellow shadows) [13]. Under 33.40 *μ*g/L DM exposure (Figure 1(b)), behavior strength showed a sudden decrease after 2 h, and it reached less than 0.4 in the 7th h (blue shadows). After 2 behavior adjustments from 8th to 16th h, behavior strength further decreased to 0 (red shadows). Under 19.66 *μ*g/L MT exposure (Figure 1(c)), behavior strength showed a slight decline, and the behavior adjustment after 3 h was not as strong as in the 13.36 *μ*g/L DM exposure (yellow shadows). Under 49.15 *μ*g/L MT exposure (Figure 1(d)), the behavior response trends were similar to the exposure of 19.66 *μ*g/L MT, but on the other hand behavior strength was much lower.

Some difference could be observed in different treatments: First, the number of adjustments in a lower concentration exposure was more than in a higher concentration exposure, and sometimes the recovery behavior strength in following adjustments might be much greater than previous ones, for example, 21 h later in 13.36 *μ*g/L DM exposure. Second, the behavior adjustment in DM was more evident than in MT exposure, specifically in a lower concentration exposure; this may be partly due to the difference in toxic mechanisms. The toxic effects of DM on organisms by combining with the Na^+^ channel on the cell membrane made the number of open Na^+^ channels increase [63]. For MT, inhibiting the activity of AChE made the AChE loses its normal physiological activity which leads to the disorder of ACh metabolism and blocks the normal conduction of the nervous system [64]. These results suggest that behavior homeostasis did exist for* D. magna* under environmental stress. Meanwhile, these results are consistent with previous results, which advised evident stepwise behavior responses including no effect, stimulation, acclimation, adjustment (readjustment), and toxic effect [13]. For example, in 33.40 *μ*g/L DM treatment, the acclimation phase occurred in 3 h, adjustment started in 8 h, and the toxic effect phase occurred in 16 h. In 49.15 *μ*g/L MT treatment, acclimation begins at 1 h, adjustment phase begins at 18 h, and at 21 h the toxic effect occurred.

Similar to the results found in swimming behavior, AChE activity (% of control) of* D. magna* in both DM and MT exposure showed evident inhibition. In the treatments of DM (Figures 1(a) and 1(b)), the AChE inhibition trends in both 13.36 *μ*g/L and 33.40 *μ*g/L were similar. At the beginning of the exposure, AChE activity increased to more than 105%; then the inhibition occurred with some recovery. AChE activity was almost as its lowest type at the end of each treatment; obviously in dead individuals, it was much lower than live ones under the same exposure time. However, there were some differences: (i) 50% inhibition of AChE activity occurred about 4 h earlier in 33.40 *μ*g/L than in 13.36 *μ*g/L DM; (ii) the ability of AChE activity recovery was much higher in lower concentration treatments. In the treatments of MT (Figures 1(c) and 1(d)), AChE inhibition differed in 19.66 *μ*g/L compared with that in 49.15 *μ*g/L. In lower concentration treatments, AChE activity was slowly decreasing with a recovery at about 18 h, and the levels were constant at above 50% during the 24-hour period. AChE activity was comparatively lower than 50% after 6 h in a higher concentration with several recoveries. The standard deviation in Figure 1 showed the vibration amplitude of both behavior strength and the AChE activity, and it suggested that the vibration amplitude was different in different behavior responses according to the criteria for different stages based on the Stepwise Behavior Response Model (Table 3): it is bigger in both acclimation and adjustment than in no effects-stimulation and toxic effects.

After the first period (approximately hour 2 to hour 6), there were effects on AChE activity in different treatments; significant inhibition with *p* < 0.05 occurred in all the treatments with several recoveries. Meanwhile, AChE activity in DM was easily inhibited, which suggests that some difference in the toxic mechanisms might induce these results. It is clear that pyrethroid insecticides possess inhibitory effects on AChE activity of* D. magna* and carbamates, which could exert their toxic effect by inducing oxidative stress with an alteration in enzymatic and nonenzymatic antioxidant systems [27, 29].

These results suggest that AChE activity might increase at the beginning of the exposure, and it was obviously lower in dead individuals than in the live individuals. AChE activity inhibition showed that higher recovery degree happened in higher concentration treatments. Though continuous detection of AChE activity of test organisms in different treatments did not attract much attention in previous research, AChE activity of* D. magna* was similar to the previous results [35]. It showed positive effects on AChE activity at the beginning of the DM exposure and some evident recovery of AChE activity occurred during the exposure in different treatments. The reasons for the positive effects and the activity recovery might be that these insecticides stimulated the de novo synthesis of AChE in* D. magna* [65]; these effects were similarly observed in three ridge mussels* (A. plicata)* [66].

### 3.3. Correlation Analysis of Both Swimming Behavior and AChE Activity

The swimming behavior of* D. magna* in different insecticides treatments showed that the fluctuating environmental stress would indeed induce evident behavior responses, including no effect, stimulation, acclimation, adjustment (readjustment), and toxic effect. The behavior homeostasis depends on the adjustments made under environmental stress; this provides a perfect method for individuals to develop wider tolerance ability to environmental stress. It is shown that physiological responses might be important for individuals to overcome these stresses [43]. Some results of the relationship between behavior responses and AChE activity in different treatments could be observed in Figure 1: First, the tendency of both behavior strength and AChE activity decreased, but there were some differences in each treatment. Second, the values of both behavior strength and AChE activity were higher in lower concentrations (Figures 1(a) and 1(c)) than in higher concentrations (Figures 1(b) and 1(d)) at the end of the exposure. All of these results suggest that the behavior response of* D. magna* is similar to the inhibition of AChE activity in those treatments. 50% inhibition of AChE activity would induce an evident decrease in behavior strength due toxic effects, especially in higher concentration treatments.

**Figure 2:**
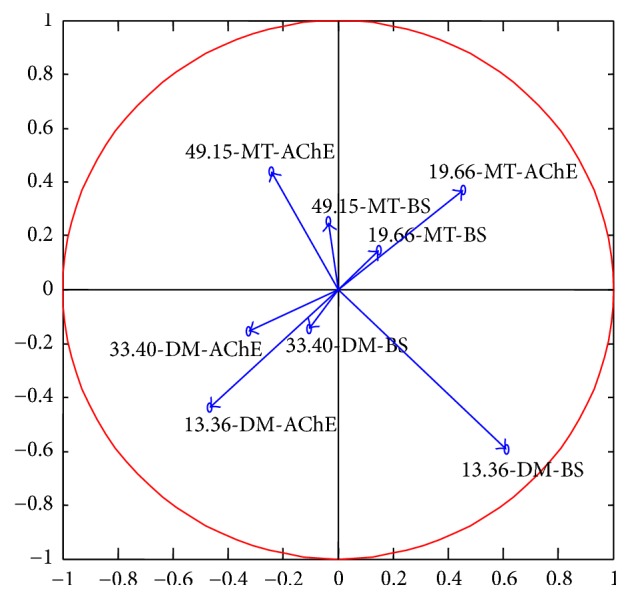
The correlation analysis of swimming behavior and AChE activity of* D. magna* based on Principal Component Analysis.

Principal Component Analysis is a statistical procedure that uses orthogonal transformation to convert a set of observations of possibly correlated variables into a set of values of linearly uncorrelated variables called principal components [67]. Principal Component Analysis is more suitable than analysis of variance for the modeling of response data, and the results of the analysis depend on the scaling of the matrix [68]. Based on the analysis of the observed results as shown in Figure 1, Principal Component Analysis in Self-Organizing Map was applied to construct a circle correlation analysis of the relationship between behavior responses and AChE activity (Figure 2).

The correlation circle results indicate that the changes in behavior strength show a positive relationship with AChE activity in each treatment. In most treatments, both behavior strength and corresponding AChE activity inhibition of* D. magna* stayed within the same field of the correlation circle. For example, the results of both parameters in 19.66 *μ*g/L and 49.15 *μ*g/L MT and 33.40 *μ*g/L DM exposure were in the upper-right, upper-left, and lower-left corner, respectively. Though behavior strength in 13.36 *μ*g/L DM exposure stayed in the lower-right corner of the circle, AChE activity inhibition was in lower-left area. The reason for this might be due to the fact that behavior homeostasis (behavior adjustment) in this treatment was stronger than in others. The results of both behavior strength and AChE activity in different treatments (Figure 1) indicated that the behavior responses were similar to each other, which included no effect, stimulation, acclimation, adjustment (readjustment), and toxic effect [13]. As reported by Xuereb et al. [34], the loss of the nerve conduction ability correlated with AChE activity inhibition played a significant role in the process.

Table 2 shows the Kalman filtering linear regression analysis of both whole-body AChE activity (% of control) and behavior strength of* D. magna* in different treatments with 95% confidence bounds. The results showed higher concentration treatments (33.40 *μ*g/L DM and 49.15 *μ*g/L MT) could induce a higher slope, which was more than 0.03 in the equations for both parameters. In lower concentration treatments, the slope in the equations was less than 0.02, except for the 13.36 *μ*g/L DM treatment, in which the slope in the equation was more than 0.03; this might induce the correlation analysis results in Figure 2. The *y*-intercept in the linear regression equations of both parameters did not show evident difference among different treatments. These results suggested that both circle correlation and linear regression analysis are not accurate enough to support the positive relationship between both parameters.

To illustrate the effects more clearly, both parameters were compared after one-way analysis of variance as shown in Table 3. The criteria of different swimming behavior were based on the stepwise behavior responses [13]. The results advised that all swimming behavior was regarded as normal (no effects) in control and AChE activity data of* D. magna* kept approximately 97% with 0.79 behavior strength. During no effects and stimulation, the behavior strength in all of the treatments kept constant, which was 0.79. There was some difference in AChE activity, which showed a significant difference with *p* < 0.05 in both higher concentration treatments (33.40 *μ*g/L DM and 49.15 *μ*g/L MT); furthermore, it was higher in 33.40 *μ*g/L DM than in control but lower in 49.15 *μ*g/L MT than in control. The increase of AChE activity in 33.40 *μ*g/L DM suggests an induction effect in the first exposure period, which was also observed by Xuereb et al. [34, 43]. Comparing these to the control results, both the two parameters in the other behavior responses showed significant difference (*p* < 0.01), which revealed an extreme behavior decrease and AChE activity inhibition. The results in Table 3 also illustrate that the behavior homeostasis in lower concentration treatments (13.36 *μ*g/L DM and 19.66 *μ*g/L MT) depended mainly on adjustment, which started from the 11th hour of exposure where no toxic effects occurred.

Previous research on the relationship between AChE activity levels and behavior homeostasis has illustrated that AChE activity inhibition resulted in unregulated nerve ending activation and paralysis in organisms and could induce abnormal behavior responses [31]. Our observations showed that a 50% activity decrease of AChE may cause the toxic effect of swimming behavior, and fluctuating environmental stress may cause an induction effect of AChE activity at times. Though the de novo synthesis of AChE in* D. magna* might help AChE activity recover, the trends during the 24 h exposure in different treatments were downward. These results clearly showed that the environmental stress caused by both DM and MT inhibits AChE activity and then induces a stepwise behavior response, though the toxic effects of these insecticides act as indirect and direct inhibitors of AChE separately [34]. Therefore, it can be ascertained that AChE activity inhibition is part of the intrinsic response mechanism of behavior homeostasis based on behavior strength.

## 4. Conclusion

This study demonstrates the role of AChE in the behavior homeostasis of* D. magna* under both DM and MT stress. Differences were registered in different treatments based on behavior strength and AChE activity. Such results confirmed that AChE could be used as one of the biomarkers for both indirect and direct inhibitors, and a clear stepwise behavior response could be induced by these inhibitors. Meanwhile, 50% AChE inhibition may cause the toxic effect of swimming behavior in a fluctuating environment, which clarifies that AChE activity inhibition is part of the intrinsic response mechanism of behavior homeostasis based on behavior strength. Nevertheless, current data of both swimming behavior and AChE activity of one kind of individuals cannot serve as direct and thorough evidence for the key role of AChE in the behavior homeostasis. The link between AChE inhibition and acute behavior homeostasis of different chemicals to different organisms has yet to be analyzed and will be established in future studies.

## Figures and Tables

**Table 1:** The 24 h acute toxic effects of both DM and MT on *D. magna*.

Chemicals	LC_50_ (*μ*g/L)	95% confidence interval (*μ*g/L)	Equation of linear regression	*r*
DM	6.68	6.441–7.026	*Y* = 2.026*X* + 15.485	0.992
MT	9.83	9.148–10.184	*Y* = 3.411*X* + 22.081	0.984

**Table 2:** Linear regression analysis of both whole-body AChE activity (% of control) and behavior strength of *D. magna* in different treatments with 95% confidence bounds.

Treatments	BS			AChE		
	Linear regression equation	Slope	*y*-intercept	Linear regression equation	slope	*y*-intercept
13.36 *μ*g/L DM	*Y* = −0.016*X* + 0.722	(−0.027, −0.006)	(0.581, 0.863)	*Y* = −0.034*X* + 1.082	(−0.044, −0.023)	(0.934, 1.229)
33.40 *μ*g/L DM	*Y* = −0.030*X* + 0.732	(−0.035, −0.026)	(0.665, 0.799)	*Y* = −0.031*X* + 0.862	(−0.040, −0.023)	(0.748, 0.976)
19.66 *μ*g/L MT	*Y* = −0.018*X* + 0.766	(−0.021, −0.014)	(0.718, 0.813)	*Y* = −0.011*X* + 0.915	(−0.017, −0.005)	(0.833, 0.997)
49.15 *μ*g/L MT	*Y* = −0.039*X* + 0.776	(−0.043, −0.034)	(0.715, 0.838)	*Y* = −0.049*X* + 1.155	(−0.058, −0.040)	(1.032, 1.277)

The slope values and the *y*-intercept values are shown with 95% confidence bounds.

**Table 3:** Statistical analysis of whole-body AChE activity (% of control) and behavior strength in different behavior responses.

Treatments	No effects and stimulation^#1^			Acclimation^#2^			(Re)adjustment^#3^			Toxic effect^#4^		
	Start time (h)	AChE activity	Behavior strength	Start time (h)	AChE activity	Behavior strength	Start time (h)	AChE activity	Behavior strength	Start time (h)	AChE activity	Behavior strength
13.3 *μ*g/L-DM	0.0	95 ± 12	0.79 ± 0.03	6.0	82 ± 9^*∗∗*^	0.46 ± 0.22^*∗∗*^	11.0	49 ± 28^*∗∗*^	0.42 ± 0.19^*∗∗*^	/	/	/
33.40 *μ*g/L-DM	0.0	102 ± 2^*∗*^	0.79 ± 0.02	3.0	95 ± 5	0.58 ± 0.14^*∗∗*^	8.0	63 ± 27^*∗∗*^	0.31 ± 0.14^*∗∗*^	16.0	18 ± 17^*∗∗*^	0.03 ± 0.03^*∗∗*^
19.66 *μ*g/L-MT	0.0	94 ± 4	0.78 ± 0.03	5.0	78 ± 6^*∗∗*^	0.57 ± 0.09^*∗∗*^	11.0	71 ± 7^*∗∗*^	0.46 ± 0.07^*∗∗*^	/	/	/
49.15 *μ*g/L-MT	0.0	92 ± 11^*∗*^	0.79 ± 0.01	1.0	58 ± 16^*∗∗*^	0.44 ± 0.19^*∗∗*^	18.0	34 ± 17^*∗∗*^	0.25 ± 0.16^*∗∗*^	21.0	20 ± 3^*∗∗*^	0.27 ± 0.18^*∗∗*^
Control	0.0	97 ± 3	0.79 ± 0.04	/	/	/	/	/	/	/	/	/

Compared with control ^*∗*^*p* < 0.05 and ^*∗∗*^*p* < 0.01; ^#^criteria for different stages based on the Stepwise Behavior Response Model: ^1^exposure period before the first significant decrease of behavior strength (SD-BS) occurred (20% [13]); ^2^after the first SD-BS until the first adjustment (20% behavior strength increase); ^3^from the first adjustment to toxic effects; ^4^starting from the time when behavior strength of *D. magna* is lower than 0.2 and there is no recovery. AChE activity data of immovable individuals in different treatments were not shown in the table.
